# Genomic characterization of patient-derived xenograft models established from fine needle aspirate biopsies of a primary pancreatic ductal adenocarcinoma and from patient-matched metastatic sites

**DOI:** 10.18632/oncotarget.7718

**Published:** 2016-02-25

**Authors:** Robert J. Allaway, Dawn A. Fischer, Francine B. de Abreu, Timothy B. Gardner, Stuart R. Gordon, Richard J. Barth, Thomas A. Colacchio, Matthew Wood, Balint Z. Kacsoh, Stephanie J. Bouley, Jingxuan Cui, Joanna Hamilton, Jungbin A. Choi, Joshua T. Lange, Jason D. Peterson, Vijayalakshmi Padmanabhan, Craig R. Tomlinson, Gregory J. Tsongalis, Arief A. Suriawinata, Casey S. Greene, Yolanda Sanchez, Kerrington D. Smith

**Affiliations:** ^1^ Department of Pharmacology and Toxicology, Geisel School of Medicine, Dartmouth College, Hanover, NH 03755, USA; ^2^ Department of Surgery, Division of Surgical Oncology, Dartmouth-Hitchcock Medical Center, Lebanon, NH 03756, USA; ^3^ Department of Pathology, Dartmouth-Hitchcock Medical Center, Lebanon, NH 03756, USA; ^4^ Department of Medicine, Section of Gastroenterology and Hepatology, Dartmouth-Hitchcock Medical Center, Lebanon, NH 03756, USA; ^5^ Dartmouth-Hitchcock Norris Cotton Cancer Center, Lebanon, NH 03756, USA; ^6^ Department of Genetics, Geisel School of Medicine, Dartmouth College, Hanover, NH 03756, USA; ^7^ Department of Medicine, Dartmouth-Hitchcock Medical Center, Lebanon, NH 03756, USA; ^8^ Institute for Quantitative Biomedical Sciences, Dartmouth College, Hanover, NH 03755, USA; ^9^ Current location: Department of Pathology, University of California, San Francisco, CA 94143, USA

**Keywords:** patient-derived xenograft, fine needle aspirate biopsy, pancreatic ductal adenocarcinoma, KRAS, CDK9

## Abstract

N-of-1 trials target actionable mutations, yet such approaches do not test genomically-informed therapies in patient tumor models prior to patient treatment. To address this, we developed patient-derived xenograft (PDX) models from fine needle aspiration (FNA) biopsies (FNA-PDX) obtained from primary pancreatic ductal adenocarcinoma (PDAC) at the time of diagnosis. Here, we characterize PDX models established from one primary and two metastatic sites of one patient. We identified an activating *KRAS* G12R mutation among other mutations in these models. In explant cells derived from these PDX tumor models with a *KRAS* G12R mutation, treatment with inhibitors of CDKs (including CDK9) reduced phosphorylation of a marker of CDK9 activity (phospho-RNAPII CTD Ser2/5) and reduced viability/growth of explant cells derived from PDAC PDX models. Similarly, a CDK inhibitor reduced phospho-RNAPII CTD Ser2/5, increased apoptosis, and inhibited tumor growth in FNA-PDX and patient-matched metastatic-PDX models. In summary, PDX models can be constructed from FNA biopsies of PDAC which in turn can enable genomic characterization and identification of potential therapies.

## INTRODUCTION

Precision medicine (using a patient's unique genetic and molecular data to inform treatment, also known as personalized medicine) may present clinicians with opportunities to improve patient care. Ideally, data regarding a patient's tumor could be used in a predictive manner to inform the clinician of the most appropriate therapy. However, there are serious challenges in translating molecular and genetic data into clinical practice. Current clinical trials of targeted therapeutics focus on performance in a patient cohort and cannot evaluate multiple therapeutic options for individual patients. To advance precision medicine that benefits individuals, we must develop robust models to evaluate the efficacy of potential therapies while the patient is alive.

Patient-derived xenograft (PDX) models address this unmet need by allowing clinicians to evaluate and compare multiple therapies. The standard approach to construct PDX models relies on surgical specimens and is well characterized in pancreatic ductal adenocarcinoma (PDAC). [1,2] Such models recapitulate the heterogeneous tumor morphology, response to chemotherapeutics, and gene expression patterns observed in patient tumors. [1,3–9] Engrafting tumors in these models relies upon factors such as tumor type, cell number, murine host strain, protocol methodology, and tumor biology. [1,10–12] In published research, the rates of engraftment and tumor growth range from 14 to 21 weeks. [1,4,12] In clinical practice, the short window between diagnosis and disease progression for aggressive malignancies limits the application of PDX models from surgical specimens as a method to evaluate individualized therapies. [13,14] While technically feasible, construction of PDX models with surgical specimens from aggressive malignancies is limited to patients with localized tumors that can be surgically removed. This is especially true for pancreatic cancer which is characterized by late presentation and only a minority (10-15%) of patients present with localized tumors that are amenable to up-front surgical resection.

One way to address this problem is to establish PDX models from patients with early-stage disease at the time of diagnosis. For many cancers, a tissue diagnosis is established by fine needle aspiration (FNA) biopsy. This specimen could be used to establish FNA-PDX models from early-stage disease. At our institution, patients undergo an endoscopic ultrasound (EUS) with FNA biopsy to establish a diagnosis of PDAC before the patient is considered for neoadjuvant therapy and resection. This biopsy method allows multiple quadrants within the primary tumor to be targeted for tumor cell acquisition. The resulting pooled specimen represents random sampling of tumor cells from the primary tumor microenvironment. After tissue diagnosis is established, all patients in our cohort are enrolled onto neoadjuvant chemoradiation protocols. Therefore, our clinical pathway does not allow us to obtain treatment naïve tumor tissue for genomic characterization or PDX engraftment at the time of surgical resection.

Unfortunately, FNA specimens derived from primary tumors such as PDAC contain a paucity of tumor cells mixed with host stromal cells. The FNA specimen therefore often contains too few tumor cells to perform genomic profiling. However, PDX tumors derived from FNA biopsies provide sufficient material for genomic characterization. [15] Using targeted sequencing, we observed that the mutational profile of FNA-PDX tumors matches that of models derived from patient-matched peritoneal and liver metastases. Genomic characterization of these models revealed a *KRAS* mutation. Increased RAS pathway signaling by oncogenic *KRAS* mutations has been implicated as a driver in PDAC; mutations in *KRAS* are found in over 90% of pancreatic tumors. [16,17] Direct and effective targeting of mutant KRAS in tumors has thus far not been achieved. [18,19] To circumvent this challenge, we previously set out to find the Achilles heel of cells with mutations in the RAS pathway. The principles of synthetic lethality allow us to treat a tumor with minimal toxicity to non-cancerous cells by exploiting vulnerabilities caused by oncogenic alterations. [20–23] Indeed, this approach has previously been utilized to discover synthetic lethal interactions in *KRAS*-mutant cells. [24] We devised an approach to identify compounds and experimental drugs that are synthetic lethal with increased RAS signaling caused by loss of the RAS-GTPase activating protein (RAS-GAP) NF1. [25] One of our tool compounds identified in our synthetic lethality screen shares a target (cyclin dependent kinase 9, CDK9) with two drugs in clinical trials. [25–27] Here, we show that FNA-PDX and patient matched metastatic-PDX models with *KRAS* mutations are sensitive to inhibitors of CDK9.

This proof of concept study demonstrates that FNA-PDX tumor models can be used to evaluate personalized therapies such as CDK inhibitors for rapidly progressing malignancies like pancreatic adenocarcinoma. There are several challenges for PDX-directed precision medicine. The models must be established during the clinical window for aggressive malignancies, they must capture the clones present within the primary tumor responsible for recurrence, and they must allow us to compare therapies before recurrence. Here, we describe an FNA-PDX protocol that addresses these challenges.

## RESULTS

### FNA-PDX models are efficiently engrafted from FNA specimens at the time of diagnosis prior to planned neoadjuvant therapy

**Figure 1:**
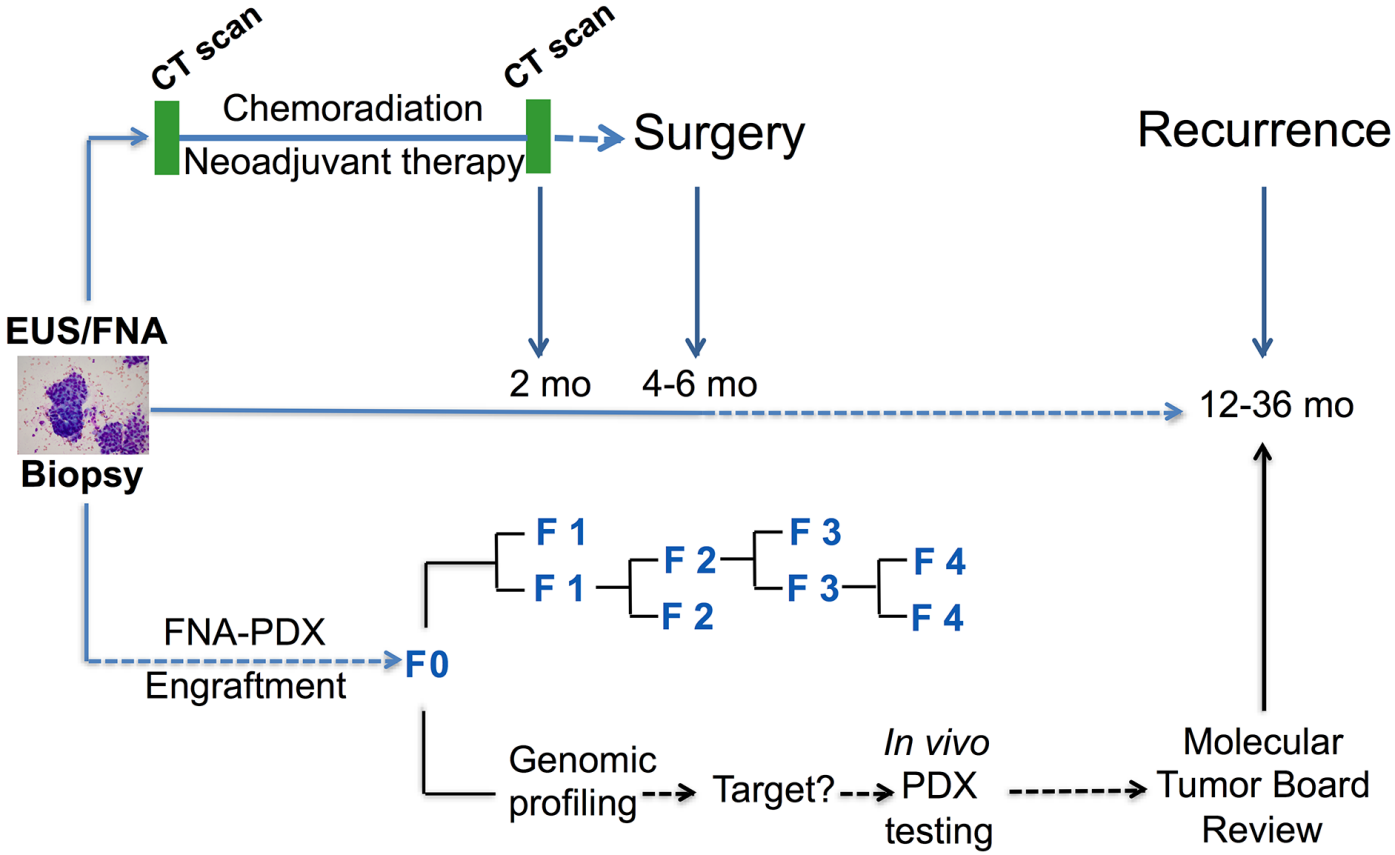
Clinical pathway for establishing FNA-PDX models at time of diagnosis of pancreatic ductal adenocarcinoma Patients enrolled in this study undergo an EUS-FNA biopsy to establish a diagnosis of PDAC prior to consideration of neoadjuvant chemotherapy and resection. During this procedure, a pooled FNA specimen is obtained for engraftment into an NSG mouse. This F0 FNA-PDX model is genomically characterized and passaged to further mice to enable evaluation of multiple therapies. Simultaneous to the development of the FNA-PDX model, patients with a diagnosis of PDAC in our cohort are enrolled onto neoadjuvant therapy protocols with subsequent resection of the tumor.

From December 2011 until May 2014, 34 patients were consented for FNA-PDX engraftment. 29 patient FNA specimens were acquired and engrafted into a subcutaneous flank pocket of a single NOD.Cg-*Prkdc^scid^*Il2rg*^tm1Wjl^*/SzJ (NSG) male mouse. Three patients had non-PDAC histology on final cytopathologic diagnosis and two patients had metastatic disease diagnosed at the time of EUS resulting in 24 patient FNA specimens engrafted from patients with localized biopsy proven PDAC. The specimen was placed directly into a cryovial on ice. The warm ischemia time, defined as the time from FNA biopsy until placement onto ice, was negligible. The median cold ischemia time (the time from biopsy until the time of mouse implantation) was 37 minutes. For patients who underwent subsequent surgery, tumor tissue was acquired from either primary tumors or metastatic sites for PDX engraftment. The clinical protocol and FNA-PDX program are illustrated in Figure 1.

To date, 9/24 (37.5%) FNA specimens have been successfully engrafted as PDX tumors. For patients who were discovered to have metastatic disease upon surgical exploration, tumor tissue was acquired and 10/10 (100%) of representative metastatic sites were successfully engrafted. Once engrafted, all but one FNA-PDX primary and metastatic-PDX models were successfully passaged up to the fifth (F5) passage. Once established, frozen PDX tumors could be thawed and engrafted for *in vivo* testing of therapeutic response.

### FNA-PDX and patient matched metastatic-PDX tumor morphology and growth rates

We selected patient #008's FNA-PDX as a proof of concept for model characterization. This patient is unique because metastatic disease developed during the time of FNA-PDX engraftment. Two metastatic sites of disease (peritoneal carcinomatosis and liver metastases) were biopsied and engrafted as metastatic M1 and M2 PDX models, respectively. This allowed us to characterize FNA and metastatic-derived PDX models concurrently.

**Figure 2:**
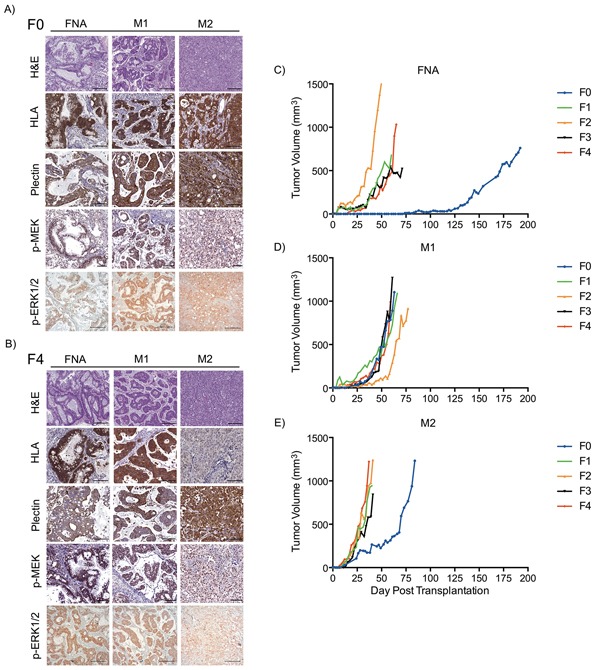
FNA-PDX and metastatic-PDX tumor growth and morphology **A-B.** Representative images of H&E staining and immunohistochemical expression of human HLA (a marker specific to cells of human origin), plectin-1 (a marker of PDAC cells), phosphorylated MEK1/2 and phosphorylated ERK1/2 (markers of RAS pathway activity) in FNA-PDX and metastatic-PDX tumors for passages F0 (A) and F4 (B). Scale bars: 200 (H&E, p-ERK) μm and 100 μm (HLA plectin-1, p-MEK). **C-E.** FNA-PDX and patient-matched metastatic-PDX tumor morphology and growth rates of successive passaged tumors.

The FNA-PDX tumor resembled the morphology, glandular formation, differentiation, and the desmoplastic stroma commonly seen in PDAC. We observed this in both the F0 (Figure 2A) and F4 (Figure 2B) passages. The morphologic heterogeneity between the FNA-PDX and the two metastatic models is depicted by H&E staining. The tumor contained mouse stromal cells (negative by human HLA immunohistochemical staining) recruited by the engrafted tumor cells (positive by human HLA immunohistochemical staining). The human tumor cells retained the expression of plectin-1, a putative PDAC biomarker (Figure 2A, B). [28] The RAS/MAPK signaling pathway was active, as evidenced by the immunohistochemical expression of the downstream effectors phosphorylated MEK and phosphorylated ERK1/2 (Figure 2A, 2B).

Tumor engraftment and growth characteristics of the FNA-PDX and patient-matched metastatic M1 and M2 models are shown in Figure 2C-E. The FNA-PDX F0 tumor took nearly 18 weeks to reach 5 mm in diameter, while passaged F1-F4 tumors uniformly grew faster (Figure 2C). Interestingly, the passaged FNA-PDX tumors growth rates were similar to the patient-matched passaged metastatic M1 (Figure 2D) and M2 (Figure 2E) models.

### The FNA-PDX pipeline stably maintains primary tumor clones that resemble PDX tumors established from sites of metastases

**Figure 3:**
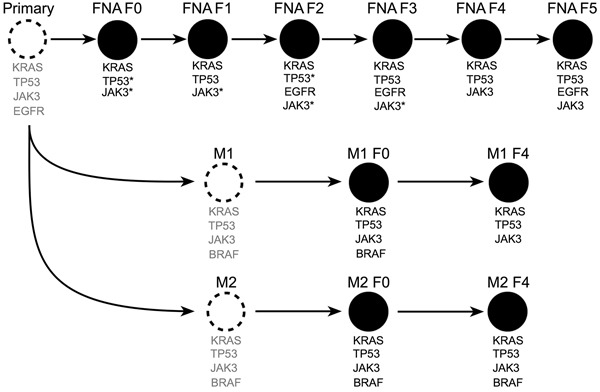
Next-generation sequencing of passaged FNA-PDX models revealed shared alleles with metastatic sites Unobserved tumors (dashed circles) with inferred alleles (grey text) based on sequencing of F0-F5 primary and F0 & F4 metastatic tumors (black filled circles). Mutations identified as unique to later passage PDX models from the primary tumor are present in early passage PDX models from metastatic sites. This suggests that passaging of FNA-PDX models selects for tumor cells that match the genetic profile of the metastatic tumors. *did not meet quality thresholds for NGS and were confirmed by SNaPshot genotyping. **amplicon drop-out by NGS, confirmed by SNaPshot.

We used a targeted next-generation sequencing (NGS) panel that consisted of 48 cancer genes in the F0-F5 passages of the patient #008 FNA-PDX model (Table 1, Figure 3). Variants detected were then confirmed by a SNaPshot genotyping assay (Table 1). Genomic characterization of an FNA biopsy is generally limited by the paucity of tumor cells. For this patient there was not enough FNA specimen available after the clinical diagnostic procedures to perform genomic characterization. Therefore, we characterized tissue obtained from the FNA-PDX model as a surrogate of the primary tumor. In the FNA-PDX F0 and F1 passage, we identified three variants. One was a *TP53* mutation (Y220C; COSM10758) in which inactivating mutations have been observed following *KRAS* activating mutations in PDACs. [29,30] The *TP53* mutation had low coverage, however it was confirmed with a SNaPshot assay and Sanger sequencing. The other mutation was a *KRAS* mutation (G12R; COSM518) that has been frequently observed in PDACs. [31] This *KRAS* allele is an indicator of poor prognosis in PDACs. [32] A *JAK3* mutation (V722I; COSM34213) was also observed in the F0 FNA-PDX model. This mutation in *JAK3* has not been reported in PDAC but has been observed in acute megakaryoblastic leukemia. [33,34] Due to a limited FNA biopsy sample size, sequencing of the primary tumor was impossible. Inferred mutations in the patient tumor in Figure 3 are labeled in grey text.

**Table 1:** Summary of next-generation sequencing of 50 cancer genes from the initial F0 and late F4 passages of FNA-PDX #008 (FNA) and patient-matched metastatic-PDX (M1/M2) models Alleles that were detected but eliminated from NGS data due to read quality were subsequently validated with a SNaPshot genotyping approach. Italicized variants were detected at an allelic frequency < 10%.

Sample	Gene	Nucleotide Change	AA Change	COSMIC ID	NGS?	SNaPshot?
FNA F0	KRAS	c.34G>C	G12R	COSM518	Yes	Yes
	TP53	c.659A>G	Y220C	COSM10758	Yes*	Yes
	JAK3	c.2164G>A	V722I	COSM34213	No**	Yes
FNA F1	KRAS	c.34G>C	G12R	COSM518	Yes	Yes
	TP53	c.659A>G	Y220C	COSM10758	Yes	Yes
	JAK3	c.2164G>A	V722I	COSM34213	Yes*	Yes
FNA F2	KRAS	c.34G>C	G12R	COSM518	Yes	Yes
	TP53	c.659A>G	Y220C	COSM10758	Yes	Yes
	JAK3	c.2164G>A	V722I	COSM34213	Yes*	Yes
	*EGFR*	*c.2219_2220insCATCG*	*I740_P741insHR*		Yes	No
	*EGFR*	*c.2236_2250delGAATTAAGAGAAGCA*	*E746_A750delELREA*	*COSM6225*	Yes	No
FNA F3	KRAS	c.34G>C	G12R	COSM518	Yes	Yes
	TP53	c.659A>G	Y220C	COSM10758	Yes	Yes
	JAK3	c.2164G>A	V722I	COSM34213	Yes	Yes
	*EGFR*	*c.2219_2220insCATCG*	*I740_P741insHR*		Yes	No
	*EGFR*	*c.2236_2250delGAATTAAGAGAAGCA*	*E746_A750delELREA*	*COSM6225*	Yes	No
FNA F4	KRAS	c.34G>C	G12R	COSM518	Yes	Yes
	TP53	c.659A>G	Y220C	COSM10758	Yes	Yes
	JAK3	c.2164G>A	V722I	COSM34213	Yes	Yes
FNA F5	KRAS	c.34G>C	G12R	COSM518	Yes	Yes
	TP53	c.659A>G	Y220C	COSM10758	Yes	Yes
	JAK3	c.2164G>A	V722I	COSM34213	Yes	Yes
	*EGFR*	*c.2219_2220insCATCG*	*I740_P741insHR*		Yes	No
	*EGFR*	*c.2236_2250delGAATTAAGAGAAGCA*	*E746_A750delELREA*	*COSM6225*	Yes	No
M1 F0	KRAS	c.34G>C	G12R	COSM518	Yes	n/a
	TP53	c.659A>G	Y220C	COSM10758	Yes	n/a
	JAK3	c.2164G>A	V722I	COSM34213	Yes	n/a
	BRAF	c.1405G>C	G469R	COSM455	Yes	n/a
M1 F4	KRAS	c.34G>C	G12R	COSM518	Yes	n/a
	TP53	c.659A>G	Y220C	COSM10758	Yes	n/a
	JAK3	c.2164G>A	V722I	COSM34213	Yes	n/a
M2 F0	KRAS	c.34G>C	G12R	COSM518	Yes	n/a
	TP53	c.659A>G	Y220C	COSM10758	Yes	n/a
	JAK3	c.2164G>A	V722I	COSM34213	Yes	n/a
	BRAF	c.1405G>C	G469R	COSM455	Yes	n/a
M2 F4	KRAS	c.34G>C	G12R	COSM518	Yes	n/a
	TP53	c.659A>G	Y220C	COSM10758	Yes	n/a
	JAK3	c.2164G>A	V722I	COSM34213	Yes	n/a
	BRAF	c.1405G>C	G469R	COSM455	Yes	n/a

Sequencing the FNA-PDX F2-F5 passages indicated the presence of the same *KRAS, TP53* and *JAK3* mutations observed in passages F0 and F1. In passages F2, F3 and F5, we also observed two *EGFR* mutations (I740_P741insHR, E746_A750delELREA; COSM6225) that occurred at an allelic frequency below 10%. The *JAK3* mutation was not detected by NGS in the FNA F0 sample due to amplicon drop-out. *EGFR* mutations were not confirmed due to low allelic frequency.

Targeted NGS was also performed on the patient-matched metastatic-PDX models. Sequencing of the M1 and M2 metastatic-PDX models strongly suggests that the *KRAS, TP53* and *JAK3* mutations existed in the primary pancreatic tumor, as the same *KRAS, TP53,* and *JAK3* variants were observed in the FNA, M1, and M2 PDX models (Figure 3). Additionally, sequencing of the F0 passage of both metastases revealed a *BRAF* (G469R; COSM455) mutation. Co-mutation in *BRAF* and *KRAS* in PDAC has been reported by some groups while others have found that mutations in these two genes are mutually exclusive. [35–37] The observed *BRAF* mutation in the F0 passage of the peritoneal metastasis (M1) was not detectable in the F4 passage. In contrast, the *BRAF* mutation in the F0 from the liver metastasis (M2) was maintained through the F4 passage. There are two possibilities consistent with our data; that the variants present in the F0 FNA-PDX also existed in the primary tumor, or that they were independently acquired during engraftment of the F0 FNA-PDX as well as in the process of engraftment of the F0 M1/M2 metastatic-PDX models. We consider the former to be more likely, but do not have the data to distinguish these two possibilities.

### Low passage explants from PDX models established *in vitro* retain the same markers as the tumor cells *in vivo*

**Figure 4:**
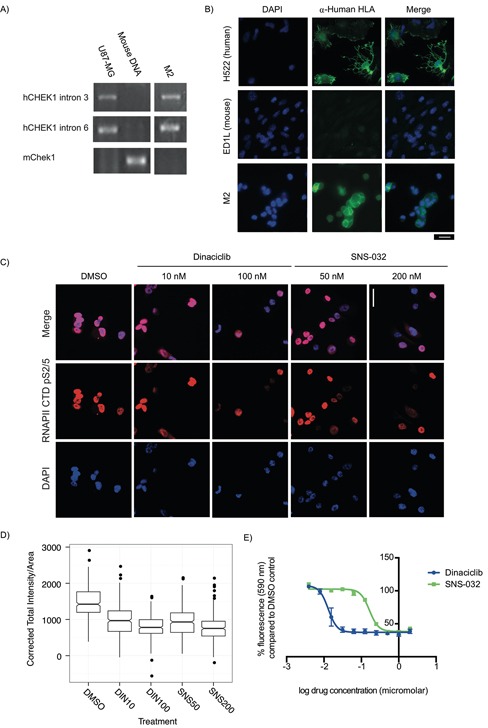
Explant cells generated from PDX models for rapid testing of targeted therapeutics are sensitive to CDK9 inhibitors **A-B.** PCR primers specific to human CHEK1 and mouse *Chek1* were used to identify mouse and human content in PDX explant cells, using U87-MG cells as a human positive control and mouse genomic DNA as a mouse positive control. (A) Cells from a liver metastasis explant (M2) had human *CHEK1* DNA but did not have mouse *Chek1* DNA. (B) To validate this, α-Human HLA class 1 A, B, and C expression (a marker of cells of human and not mouse origin) were examined by immunofluorescence. All M2 explant cells evaluated were found to be α-Human HLA A, B and/or C positive. Human (H522) and mouse (ED1L) cell lines were used as controls. Scale bar: 20 μm. **C.** A *KRAS* mutant PDX explant treated for 24 h with the CDK9 inhibitors dinaciclib (10 nM and 200 nM) and SNS-032 (50 nM and 200 nM) has reduced Ser2/5 phosphorylation of the CTD of the large subunit of RNAPII. Scale bar: 20 μm. **D.** Box plots of the quantification of total fluorescent intensity per unit area of cells treated with dinaciclib and SNS-032 and stained for p-Ser2/5 RNAPII CTD. Data presented was collected in three independent experiments. **E.** Dinaciclib (IC50: 13 nM) and SNS-032 (IC50: 165 nM) inhibit the growth of explant cells during a 3 day treatment. Data presented is the average fluorescence and corresponding standard error of the mean for three independent experiments.

To rapidly evaluate drug efficacy in tumor cells, we established explants *in vitro* from the #008 M2-PDX tumor (Figure 4A) and from xenograft tumors generated with cell cultures directly established *in vitro* from patient #008's metastatic pancreatic tumors (M1, Supplementary Figure S1). We used polymerase chain reaction (PCR) with human and mouse specific primers to determine the species composition of the explants (Figure 4A, Supplementary Figure S1A). We confirmed human content by immunofluorescence with antibodies against human leukocyte antigens (HLAs) class I A, B, and C, which are expressed by most nucleated human cells (Figure 4B, Supplementary Figures S1B). [38] These methods indicated that the explants were entirely of human origin and not mouse stromal origin. Sanger sequencing confirmed that these cells maintained the *KRAS* G12R mutation observed in the M1/M2 tumors (Supplementary Figure S2).

### CDK9 inhibition suppresses growth of primary and metastatic-PDX tumors *in vitro* and *in vivo*

We previously used high-throughput screens to identify drug targets for cancer cells in which increased RAS signaling due to loss of a RAS-GAP drives tumor formation. Using this approach we found that cells with increased RAS signaling are sensitive to inhibition of a cyclin dependent kinase that phosphorylates RNA Pol II CTD on Ser2. [25,39] One RNA Pol II CTD Ser2 kinase in humans is CDK9 (the functional subunit of p-TEFb), for which there are two inhibitors in clinical trials (dinaciclib and SNS-032). We evaluated the effect of dinaciclib and SNS-032 on phosphorylation of RNA Pol II CTD Ser2/5 in explant cells derived from patient #008's PDX models. The primary explants from our M1- and M2-PDX models exhibit RNA Pol II CTD phosphorylation at Ser2/5 that can be reduced by treatment with either dinaciclib or SNS-032 (Figure 4C, 4D, Supplementary Figure S1C, S1D). Furthermore, dinaciclib and SNS-032 stopped the *in vitro* growth/reduced viability of explants derived from the PDX models established from metastatic tumors at concentrations that significantly decreased p-RNAPII CTD Ser2/5 (Figure 4C–4E, Supplementary Figure S1E-S1G).

**Figure 5:**
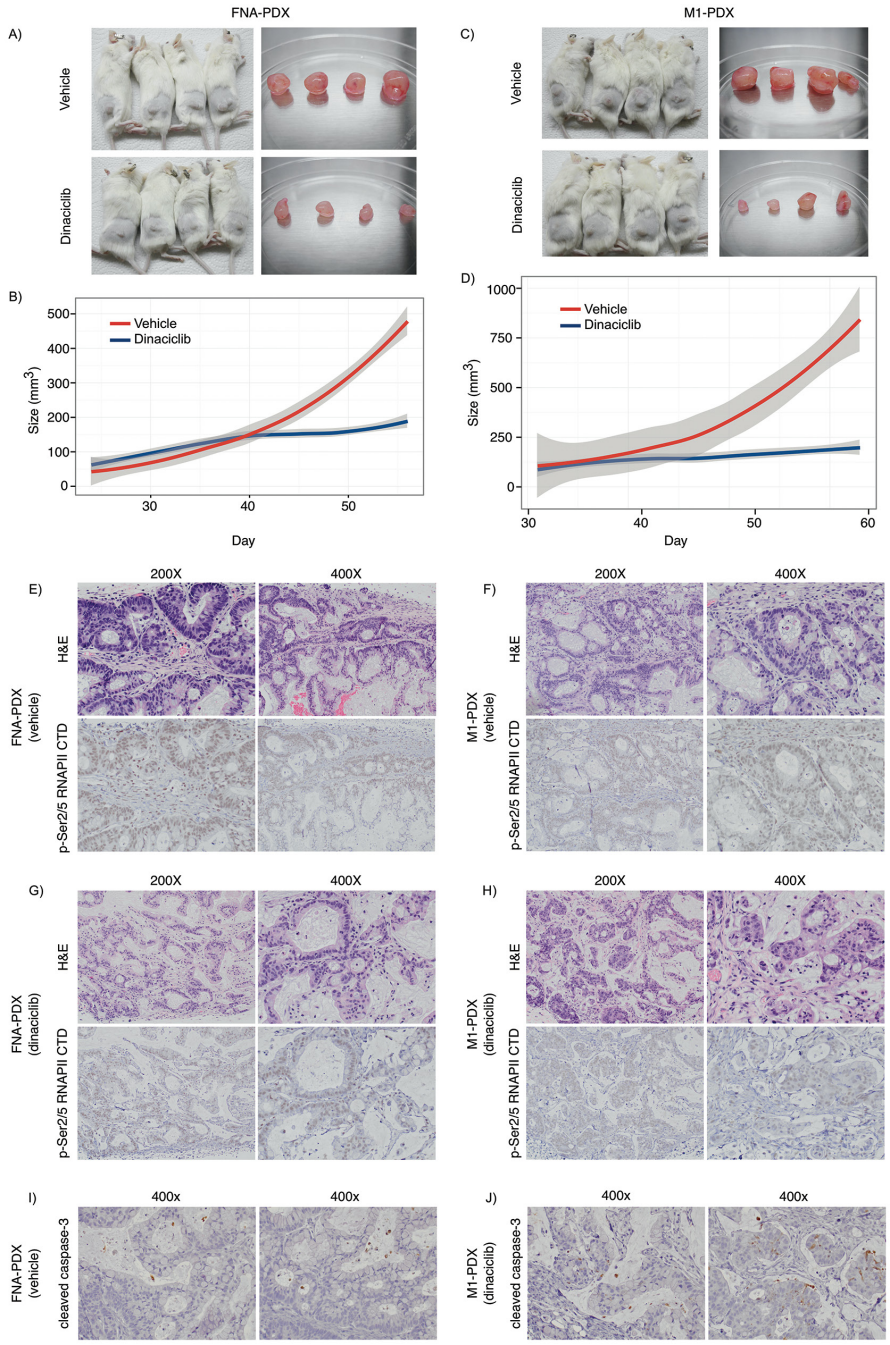
Dinaciclib inhibits tumor growth in KRAS mutant FNA-PDX and metastatic-PDX models **A-D.** Dinaciclib inhibits the growth of FNA-primary and patient-matched metastatic-PDX tumors *in vivo*. (A) FNA-PDX models established from the patient's primary tumor were treated with 40 mg/kg dinaciclib (top) or vehicle (bottom) 3 times per week for 4 weeks when the tumor reached 62.5 mm^3^. Tumors were excised. (B) Loess regression curve of the tumor growth of FNA-PDX models from (A) during treatment with vehicle (red) or dinaciclib (blue). 95% confidence intervals are indicated by the grey region. (C) PDX models established from a peritoneal metastasis (M1 from Figure 3) were treated with 40 mg/kg dinaciclib (top) or vehicle (bottom) and tumors excised after 4 weeks of treatment. (D) Loess regression curve from (C) during treatment with vehicle (circles) or dinaciclib (squares). 95% confidence intervals are indicated by the grey region. **E-F.** Representative H&E and p-RNAPII CTD Ser2/5 immunohistochemical staining of FNA-PDX tumor sections from vehicle treated mice. Tumors treated with dinaciclib exhibit reduced p-RNAPII CTD Ser2/5 as compared to the vehicle control. (G-H) Representative H&E and p-Ser2/5 RNAPII CTD immunohistochemical staining of metastatic-PDX tumor sections from dinaciclib treated mice. **I-J.** Representative cleaved caspase-3 immunohistochemical staining in FNA-PDX tumors from vehicle (I) and dinaciclib treated mice (J).

We then tested whether dinaciclib inhibited the growth of patient #008's PDX tumors *in vivo* by implanting the FNA-PDX and the metastatic (M1-PDX) tumors in mice, and treating with dinaciclib or vehicle for four weeks (Figure 5). Dinaciclib treatment suppressed the growth of the FNA-PDX and the metastatic M1-PDX tumors (Figure 5A–5D) decreased phospho-RNAPII CTD Ser2/5 signal in the tumors, and altered tumor/stromal morphology (Figure 5E–5H). Dinaciclib treatment increased apoptosis in FNA-PDX tumors as measured by caspase-3 cleavage (Figure 5I, 5J). These data suggest that cells with a KRAS G12R mutation may be vulnerable to CDK2 and/or 9 inhibition, targets shared by both dinaciclib and SNS-032.

## DISCUSSION

We constructed PDAC PDX models from FNA biopsies. In the FNA-PDX tumor, we identified molecular lesions that predicted response to targeted therapies (CDK9 inhibition) identified through high-throughput screens. CDK9 loss in yeast was previously found to be synthetic lethal with increased Ras pathway signaling resulting from loss of the RAS-GAP NF1. [25] We observed that *KRAS* mutant FNA-PDX and metastatic-PDX explant cells had phosphorylation of Ser2/5 of the large subunit of RNAPII, and phosphorylation was reduced with two CDK9 inhibitors, dinaciclib and SNS-032. Dinaciclib, an inhibitor of CDKs 1/2/5/9, was previously evaluated as a CDK5 inhibitor in PDAC PDX models from surgical specimens. [2] SNS-032 has been shown to be selective for CDKs 2/7/9 at the effective concentration used in our study. [40] These inhibitors reduced the growth of PDX explant cells *in vitro*. We observed that dinaciclib inhibited tumor growth and induced cell death in both FNA-PDX and metastatic-PDX models. Furthermore, dinaciclib inhibited CDK9 in these PDX tumors as indicated by a reduction in phosphorylation of Ser2/5 of the large subunit of RNAPII. Taken together, these results suggest that dinaciclib's effect on growth of the KRAS-mutant FNA and metastatic pancreatic PDX models is due to inhibition of CDK2 and/or CDK9.

One major challenge that limits PDX application to individualized treatments in PDAC is tissue acquisition. To date, most PDX protocols require surgery, even for localized tumors, to obtain enough tissue for engraftment. [41] However, only a minority of patients presenting with biopsy proven PDAC can be considered for up-front surgical resection. Many patients with localized tumors cannot be considered for surgical resection due to age, comorbidities and declining performance status. For aggressive cancers, such as pancreatic and lung cancer, patients often never undergo surgery before starting systemic treatment. Kim et al. established PDX models from surgically resected tumors in patients treated with neoadjuvant therapy. [9,42] However, in order for PDX models to be used to inform patient therapy, these models need to be established from primary tumor tissue before the patient's tumor is subjected to either chemotherapy or chemoradiation therapy. One source of treatment-naïve tumor tissue is the FNA biopsy performed at diagnosis of PDAC. While FNA biopsies can establish a tissue diagnosis with excellent sensitivity and specificity there is often limited cellular material available for molecular diagnostic or research studies. In the present study, we demonstrated engraftment of EUS-FNA biopsies from treatment-naïve primary pancreatic tumors into FNA-PDX models, enabling characterization and testing of therapies.

A second major challenge to using PDX models to inform therapy in aggressive malignancies is the narrow window from the time of diagnosis to disease progression. For patients with localized PDAC who undergo neoadjuvant therapy or surgery, the median time to recurrence is 1 year and 80% recur in 2 years. [43,44] We demonstrate the establishment of FNA-PDX models within this window. While the patient is receiving standard therapies, FNA-PDX models allow molecular characterization and testing of therapeutics to inform treatment options at the time of disease recurrence.

Tumor heterogeneity complicates the use of genomic assays for personalized medicine. [45–47] To establish FNA-PDX models, we targeted multiple quadrants of the tumor during the FNA biopsy for spatially diverse sampling of tumor cells within the primary tumor. [48–50] We hypothesized that engraftment of this sample would select for the clinically relevant clone in the FNA-PDX model. Other groups have demonstrated genomic stability in PDX models. [51] The stability of the model is critical if a patient's PDX model will be used to inform therapy or for drug stability. To address this issue, Tignanelli and Yeh and colleagues showed stability of mutational frequencies in late passaged colorectal cancer and pancreatic cancer PDX models. [51] We also observed that a PDX model established from an FNA biopsy was genetically stable between the F0 and F5 passages. The FNA-PDX model had genetic features that were observed in the metastatic-PDX models. This suggests that xenotransplantation of FNA-PDX tumors permits the growth of clones represented in the patient's metastatic sites. It also suggests that PDX models generated from the FNA biopsy of the primary tumor closely represents the clones with metastatic and invasive potential. Our data show that the response of the FNA-PDX to targeted therapies is recapitulated in patient-matched PDX models established from a metastatic site. These results in the FNA-PDX model may also show activity at the time of recurrence in a metastatic setting.

In conclusion, we show that FNA-PDX and patient-matched metastatic-PDX models with KRAS mutations are sensitive to CDK9 inhibitors. This work illustrates that FNA-PDX models present significant opportunities to evaluate personalized treatments before disease recurrence for aggressive and difficult-to-treat malignancies. We show that FNA-PDX models can first, be efficiently established during the clinical window for aggressive malignancies, second, capture the clones present within the primary tumor with metastatic potential, and third, provide a platform for comparing genome-driven therapies before recurrence.

## MATERIALS AND METHODS

### Establishing PDX models

Beginning in December of 2011, Velos protocol number D11129 (CPHS 23034/IACUC 11-01-05) protocol began accrual. At our institution, patients presenting with a pancreatic mass and without metastatic disease on cross sectional imaging were considered for EUS/FNA biopsy to establish a tissue diagnosis prior to multidisciplinary review and consideration for Dartmouth-Hitchcock/Norris Cotton Cancer Center neoadjuvant therapy protocols. FNA-PDX protocol consent was obtained before the procedure. Once cytopathologic diagnosis was confirmed by on-site review a 2nd or 3rd pass/repeat EUS/FNA biopsy was obtained for tumor cell acquisition. Patient demographics, EUS findings, cytopathology acquisition numbers, procedure, and specimen acquisition times were recorded. The FNA specimen was placed immediately on ice.

FNA specimen was washed with ice-cold DMEM (Hyclone, South Logan, UT, USA) containing 1% penicillin/streptomycin (Hyclone), centrifuged at 2000rpm x 2 minutes and resuspended in 50 μL DMEM. We placed an 8-12 week old male NSG mouse under isoflurane anesthesia and opened a small pocket on its right flank. The entire pooled FNA specimen was transplanted to a single NSG mouse flank as a heterotopic implantation. Briefly, through a small flank incision we created a subcutaneous pocket wherein the FNA specimen was implanted surgically. Tumor progress was measured three times weekly until the tumor reached 10 mm^2^. This initial PDX tumor was designated F0 as described by Monsma et al, Malaney et al and others. [10,52,53] The tumor was then passaged to bilateral flanks of two subsequent mice. Passaged tumors and mice were designated F1-F7, and portions of tumor tissue were banked in 10% phosphate-buffered formalin (Polysciences, Warrington, PA, USA) for paraffin embedding, liquid nitrogen, and RNAlater (Qiagen, Valencia, CA, USA). Tissue was also banked at −140°C in FBS with 10% DMSO for later use in in-vivo experiments. All mouse procedures followed Institutional Animal Care and Use Committee protocols. Mice were raised in the Transgenics and Genetic Constructs Resource at Dartmouth College.

### Immunohistochemistry

Immunohistochemistry was performed on paraffin-embedded tissue (5 μm sections). After deparaffinization in xylene and rehydration in decreasing concentrations of EtOH, slides were boiled in Epitope Retrieval Buffer (Bethyl Lab Inc., Montgomery, TX, USA) for either 20 minutes (HLA) or 30 minutes (plectin-1, p-MEK, p-RPB1 CTD S2/5). Tissue permeabilization in 0.5% Triton-X 100/1% TBS was followed by either a 0.3% peroxide block in methanol (plectin-1, p-MEK, p-RPB1 CTD S2/5) or a normal serum block (HLA). After avidin/biotin blocking (Vector Labs, Burlingame, CA, USA), slides were incubated with the primary antibody overnight at 4°C or 1 hour at RT (p-RPB1 CTD Ser 2/5). Primary antibody dilutions were: HLA Class 1 ABC (Acris Antibodies Inc, San Diego, CA; 5 μg/mL), plectin-1 [E398P] (Abcam, San Francisco, CA, USA; 1:1000) and phospho-MEK1/2 Ser221(166F8) (Cell Signaling, Danvers, MA, USA; 1:50), phospho-p44/42 MAPK (ERK1/2) Thr202/Tyr204 (Cell Signaling, 1:300) p-RPB1 CTD Ser 2/5 (Cell Signaling; 1:25-1:200). Appropriate controls were performed on tumor sections without primary antibody (data not shown).

Phospho-MAPK p44/42 antibody incubation was performed overnight. Plectin-1 and p-MEK primary antibody incubation was followed by a 30-minute incubation with the secondary antibody (RT, 1:200). The HLA secondary antibody incubation was preceded by a 10-minute peroxide block. Secondary staining for p-MAPK p44/42 and p-Rpb1 CTD Ser 2/5 was performed with SignalStain Boost Reagent, Rabbit (Cell Signaling). After a 30-minute incubation with the ABC reagent (Bethyl Lab Inc, all antibodies except p-MAPK p44/42 and p-RPB CTD S2/5), slides were developed with DAB (BD Pharmingen, San Jose, CA, USA) and counterstained with hematoxylin/Scott's bluing reagent. H&E, cleaved caspase-3, and p-RPB CTD S2/5 slides were evaluated by a GI pathologist.

### DNA extraction, next generation sequencing, and SNaPshot assay

Genomic DNA was extracted using the Gentra PureGene Kit (Qiagen, Valencia, CA, USA). Samples were quantified by Quant-iT^TM^ PicoGreen^®^ dsDNA Assay Kit (Life Technologies, Grand Island, NY, USA) before next generation sequencing. Sample preparation and sequencing was performed by a CLIA-approved molecular pathology core facility. Libraries were generated using the llumina TruSeq Amplicon Cancer Panel that consists of 212 amplicons mapped in 48 genes. At least 250 ng of DNA was used for the hybridization of oligo probes through the targeted region of genomic DNA, followed by extension and ligation, resulting in the formation of products containing the targeted regions of interest flanked by sequences required for amplification. Indices and sequence adapters were added by PCR. Finally, libraries were purified, normalized (to ensure equal representation of each sample), pooled, and sequenced on the Illumina MiSeq system. The average cluster density was 1074.9 K/mm^2^, with 91.92% of the clusters passing quality control filters. Approximately 97.7% of the clusters were ≥Q30 (Phred quality score).

Base-calling and sequence alignment to hg19 were performed using the MiSeq reporter software. VCF files were generated using the Somatic Variant Caller for the TruSeq Amplicon Cancer Panel. VCF files were then uploaded to VariantStudio v2.1.36, where variants were annotated, classified, and filtered for quality and significance. The initial step of the filtering process was to remove non-coding or low-quality variants (quality score < 100). An additional filter was then applied to remove those that were present at less than 7.5% allelic frequency or were covered at less than 300x. Remaining variants were individually interrogated using the UCSC Genome Browser GRCh37/hg19 assembly (http://genome.ucsc.edu).

In order to confirm the variants detected by NGS, we designed a genotyping panel using the SNaPshot technology. SNaPshot is a multiplex genotyping assay that consists of multiple steps, which includes a multiplexed exon specific PCR using unlabeled oligonucleotide primer sets and a multiplexed single-base primer extension using fluorescently labeled dideoxynucleotide triphosphate. For the PCR, we designed primers for the following genes: *EGFR*, *KRAS*,*JAK3*, and *TP53*. For the extension PCR, we designed a total of three primers for the point mutations and two for the INDEL. Samples were normalized to approximately 10 ng/μl and approximately 20 ng of gDNA was used for the reaction. All samples, and a positive control for each mutation were amplified with unlabeled primers for genes and subjected to a multiplexed extension primer reaction using the SNaPshot Ready Reaction Mix (Life Technologies). Capillary electrophoresis of PCR products was performed using the ABI 3500 Genetic Analyzer with POP-7TM polymer and 50 cm capillary. The genotyping results were analyzed using Applied Biosystems GeneMapper® 4.1 software.

PCR primers:

KRAS (exon 2):

Forward: TCATTATTTTTATTATAAGGCCTGCTG

Reverse: AGAATGGTCCTGCACCAGTAA

EGFR (exon 19):

Forward: GCACCATCTCACAATTGCCAGTTA

Reverse: AAAAGGTGGGCCTGAGGTTCA

TP53 (exon 6):

Forward: AGGTCAAATAAGCAGCAGGAG

Reverse: CACTGATTGCTCTTAGGTCTGG

JAK3 (exon 16):

Forward: CTCAGTGCTCACCGACAGGA

Reverse: AAAGTGGGGGTTCGGAGAC

SNaPshot primers:

KRAS (p.G12R): GACTGACTGCTCTTGCCTACGCCAC

EGFR (c.2236_2250del15, forward): CTGACTGACTG ACTGACTGTCCCGTCGCTAT-CAAG

EGFR (c.2236_2250del15, reverse): GACTGACTGACTG ACTGACTGACTGACTGAC-TGACTGACTGATTGGC TTTCGGAGATGT

TP53 (p.Y220C): GACTGACTGACTGACTGACTGACTGACTGACTAGTGTGGTGGT-GCCCT

JAK3 (p.V722I): GACTGACTGACTGACTGACTGACTGACTGACTGACTGACTGACTG-ACTGACTGACTGACTGACTGACTGAGAAGTGTTTAGTGGC

### Establishing low passage *in vitro* cell cultures from PDX models

When tumors were passaged, a separate aliquot of tumor tissue was minced in DMEM (Hyclone) containing 10% FBS (Hyclone) and 1% penicillin/streptomycin (Hyclone) and placed into a 6-well plate (Corning, Tewksbury, MA, USA) and cultured at 37°C in humidified 5% CO_2_. Media was changed every three to four days. Once cells reached 60-70% confluence, cells were trypsinized with a 0.05% trypsin (Cellgro) solution. In an attempt to isolate tumor cells from contaminating mouse stromal cells in early passage cell cultures, we monitored the trypsin incubation step under the microscope for differential adherence of cell populations. Less adherent mouse stromal cells were washed with cold PBS, aspirated and discarded to enrich the remaining adherent tumor cell population which was subsequently replated in DMEM with 10% FBS at 1-1.5^6^ cells per 175cm^2^ flask. Early passage cell cultures were tested for mycoplasma, HIV and hepatitis B and C tested prior to *in vitro* studies.

### Cell lines

Cell lines used as controls were obtained from the American Type Culture Collection (H522, U87-MG) or were a kind gift from Dr. Ethan Dmitrovsky (ED1L). Cells were cultured in RPMI 1640 (Corning, H522/ED1L) or DMEM (Corning, U87-MG) with 10% fetal bovine serum (Atlanta Biologicals, Flowery Branch, GA, USA). Cell lines were maintained at 37°C in humidified 5% CO_2_.

### Immunofluorescence

Tumor explants were cultured on poly-D-lysine coated coverslips (Neuvitro Corporation, El Monte, CA, USA) and treated with dinaciclib (Izen Biosciences Pvt Ltd, Hyderabad, India) or SNS-032 (Selleckchem, Houston, TX, USA) for 24 hours. Cells were fixed in 3.7% formaldehyde (Fisher Scientific, Pittsburgh, PA, USA) in PBS (Corning) and blocked with IF buffer (2%[v/v] goat serum, 0.2%[v/v] Triton X-100 and 0.05%[w/v] sodium azide in PBS) at RT. Ser2/5 phosphorylation of the CTD of the large subunit of RNAPII was labeled using 1:400 rabbit anti-phospho-Rpb1 CTD Ser 2/5 #4735 at room temperature for one hour (Cell Signaling) and 1:800 goat anti-rabbit F(ab’)2 DyLight 594 at room temperature for 1 hour (Thermo Scientific, Waltham, MA, USA) diluted in IF buffer. Images were acquired with a Zeiss Imager Z1 wide-field microscope equipped with a 40x 1.3 NA EC Plan-NEOFLUAR objective and Zeiss Axiovision software.

Immunofluorescence images were composited/measured using Fiji. [54] We used previously described methods to quantify fluorescent intensity. [55,56] Each measured region was defined with DAPI channel. The same size region was measured next to the cell for the background reading. Fluorescence intensity measurements were performed on cells stained for p-RPB CTD Ser2/5. For comparisons between cells, we calculated corrected total fluorescence (CTCF) for each cell, which is the integrated density of cell minus area of selected cell times mean fluorescence of background readings for that cell. We divided the value by the area of each cell to correct for cell size. This value was averaged over 100 cells for each of 3 replicates. All data are accessible via a publically available repository (doi: 10.5281/zenodo.34430). [57]

### Characterization of explant cell lines

Tumor explants were cultured on poly-D-lysine coated coverslips (Neuvitro Corporation) and allowed to adhere overnight. Cells were fixed in 3.7% formaldehyde and blocked with IF buffer at RT. Cells of human origin were labeled using 1:200 rat anti-human HLA Class I ABC #SM2012P for one hour at RT (Acris Antibodies) and 1:100 goat anti-rat FITC for one hour at RT (Sigma-Aldrich, St. Louis, MO, USA) diluted in IF buffer. Images were acquired with a Zeiss Imager Z1 wide-field microscope equipped with a 40x 1.3 NA EC Plan-NEOFLUAR objective. Images were processed and composited with Fiji. [54]

PCR was performed using primers directed towards mouse *Chek1*, human *CHK1* (introns 3 and 6) and human *KRAS*. Species specific Chek1/CHK1 primers were used to confirm that the PDX-derived explant cells were of human origin. DNA extraction was performed in lysis buffer (0.45% Nonidet P40 [Roche, Nutley, NJ, USA], 1X ThermoPol Taq buffer (New England Biolabs, Ipwich, MA, USA), and 100 μg/mL proteinase K [Roche]) at 55°C. Amplification was performed in a 20 μL reaction volume including 1 μL DNA extract, 0.5 μM forward and reverse primer mixture, 200 μM dNTPs, 0.1 unit Taq DNA Polymerase (New England Biolabs), 2 μL 10X Standard Taq Buffer (New England Biolabs), and 15 μL nuclease-free water. The thermocycler protocol was as follows: 1. (94°C, 2 minutes), 2. (94°C, 15 seconds), 3. (58°C (*CHEK1*) or 52°C (*KRAS*), 30 seconds), 4. (72°C, 1 minute, 5. (72°C, 10 minutes), with steps 2-4 repeated for 30-40 cycles. Amplified DNA and a 100 kb ladder was run on a 1.5% TBE-agar gel with ethidium bromide for 40 minutes at 100 V and visualized with UV light. Sanger sequencing of *KRAS* was performed by the Dartmouth College Molecular Biology core facility using 5 pmol *KRAS* reverse primer and 20ng *KRAS* PCR product. Sequence chromatographs were visualized using 4Peaks.

Primers:

Mouse *Chek1*:

Forward: 5′-ccacagtctcagtgaagggc-3′

Reverse: 5′-gaagaaaaagtaaaaggcatcg-3′

Human *CHEK1*, intron 3:

Forward: 5′-atgacgccttttgccaccta-3′,

Reverse: 5′-cacccctgccatgagttgat-3′

Human *CHEK1*, intron 6:

Forward: 5′-cttactgcaatgctcgctgg-3′,

Reverse: 5′-gggtaccatggctcatgtct-3′

Human KRAS [58]:

Forward: 5′-aaggtactggtggagtatttg-3′

Reverse: 5′-gtactcatgaaaatggtcagag-3′

### Response to drugs *in vitro*

To perform drug sensitivity assays, cells were plated to 96-well plates at a concentration of 5000 cells/well. After overnight incubation, medium was removed and replaced with 100 μL of medium containing 0-2000 nM CDK9 inhibitor and DMSO (to normalize DMSO concentrations) was added. Cells were incubated for 3 days with a final 3-hour incubation in 5% AlamarBlue (Thermo Scientific). The plate was scanned at an Ex/Em of 544/590 nm, and fluorescence was normalized to vehicle control wells. Dose-response curves and IC_50_s were calculated with the Prism 6 software package (GraphPad, San Diego, CA, USA) by performing a 4-parameter logistic regression with outlier exclusion analysis.

### Response to drugs *in vivo*

FNA-PDX and metastatic-PDX models were established as described above. Mice were assigned to vehicle (20% hydroxpropyl-β-cyclodextrin, Sigma-Aldrich) or dinaciclib treatment arms ensuring that both groups had an equal distribution of tumor sizes. Treatment was initiated when tumors reached approximately 62.5 mm^3^. Mice were weighed prior to treatment and treated 3 times weekly i.p. with 40 mg/kg dinaciclib in 20% hydroxypropyl-β-cyclodextrin or an equivalent amount of vehicle for 4 weeks. Mouse weight and tumor dimensions were measured prior to every treatment and volumes were calculated using the following formula:

Volume=12(L×W2)

### Statistical methods for data analyses

Box plots were constructed using ggplot2 and the R programming language. [59,60] Tumor volumes over time were modeled using LOESS regression in R. [59] This technique models tumor volume over time using local measurements. 95% confidence intervals (grey regions) were calculated using the T approximation. All data are accessible via publically available repository (http://dx.doi.org/10.5281/zenodo.34430). [57]

## SUPPLEMENTARY FIGURES



## References

[1] Rubio-Viqueira B, Jimeno A, Cusatis G, Zhang X, Iacobuzio-Donahue C, Karikari C, Shi C, Danenberg K, Danenberg P V, Kuramochi H, Tanaka K, Singh S, Salimi-Moosavi H (2006). An in vivo platform for translational drug development in pancreatic cancer. Clin Cancer Res.

[2] Feldmann G, Mishra A, Bisht S, Karikari C, Garrido-Laguna I, Rasheed Z, Ottenhof NA, Dadon T, Alvarez H, Fendrich V, Rajeshkumar N V, Matsui W, Brossart P (2011). Cyclin-dependent kinase inhibitor Dinaciclib (SCH727965) inhibits pancreatic cancer growth and progression in murine xenograft models. Cancer Biol Ther.

[3] Rubio-Viqueira B, Hidalgo M (2009). Direct in vivo xenograft tumor model for predicting chemotherapeutic drug response in cancer patients. Clin Pharmacol Ther.

[4] Kim MP, Evans DB, Wang H, Abbruzzese JL, Fleming JB, Gallick GE (2009). Generation of orthotopic and heterotopic human pancreatic cancer xenografts in immunodeficient mice. Nat Protoc.

[5] DeRose YS, Wang G, Lin Y-C, Bernard PS, Buys SS, Ebbert MTW, Factor R, Matsen C, Milash BA, Nelson E, Neumayer L, Randall RL, Stijleman IJ (2011). Tumor grafts derived from women with breast cancer authentically reflect tumor pathology, growth, metastasis and disease outcomes. Nat Med.

[6] Kabos P, Finlay-Schultz J, Li C, Kline E, Finlayson C, Wisell J, Manuel CA, Edgerton SM, Harrell JC, Elias A, Sartorius CA (2012). Patient-derived luminal breast cancer xenografts retain hormone receptor heterogeneity and help define unique estrogen-dependent gene signatures. Breast Cancer Res Treat.

[7] Zhang X, Claerhout S, Prat A, Dobrolecki LE, Petrovic I, Lai Q, Landis MD, Wiechmann L, Schiff R, Giuliano M, Wong H, Fuqua SW, Contreras A (2013). A renewable tissue resource of phenotypically stable, biologically and ethnically diverse, patient-derived human breast cancer xenograft models. Cancer Res.

[8] Kim MP, Fleming JB, Wang H, Abbruzzese JL, Choi W, Kopetz S, McConkey DJ, Evans DB, Gallick GE (2011). ALDH activity selectively defines an enhanced tumor-initiating cell population relative to CD133 expression in human pancreatic adenocarcinoma. PLoS One.

[9] Kim MP, Truty MJ, Choi W, Kang Y, Chopin-Lally X, Gallick GE, Wang H, McConkey DJ, Hwang R, Logsdon C, Abbruzzesse J, Fleming JB (2012). Molecular profiling of direct xenograft tumors established from human pancreatic adenocarcinoma after neoadjuvant therapy. Ann Surg Oncol.

[10] Tentler JJ, Tan AC, Weekes CD, Jimeno A, Leong S, Pitts TM, Arcaroli JJ, Messersmith WA, Eckhardt SG (2012). Patient-derived tumour xenografts as models for oncology drug development. Nat Rev Clin Oncol.

[11] Torphy RJ, Tignanelli CJ, Kamande JW, Moffitt RA, Herrera Loeza SG, Soper SA, Yeh JJ (2014). Circulating tumor cells as a biomarker of response to treatment in patient-derived xenograft mouse models of pancreatic adenocarcinoma. PLoS One.

[12] Quintana E, Shackleton M, Sabel MS, Fullen DR, Johnson TM, Morrison SJ (2008). Efficient tumour formation by single human melanoma cells. Nature.

[13] Garralda E, Paz K, López-Casas PP, Jones S, Katz A, Kann LM, López-Rios F, Sarno F, Al-Shahrour F, Vasquez D, Bruckheimer E, Angiuoli S V, Calles A (2014). Integrated next-generation sequencing and avatar mouse models for personalized cancer treatment. Clin Cancer Res.

[14] Katz MHG, Wang H, Fleming JB, Sun CC, Hwang RF, Wolff RA, Varadhachary G, Abbruzzese JL, Crane CH, Krishnan S, Vauthey J-N, Abdalla EK, Lee JE (2009). Long-term survival after multidisciplinary management of resected pancreatic adenocarcinoma. Ann Surg Oncol.

[15] Marshall D, Laberge JM, Firetag B, Miller T, Kerlan RK (2013). The changing face of percutaneous image-guided biopsy: molecular profiling and genomic analysis in current practice. J Vasc Interv Radiol.

[16] Biankin A V, Waddell N, Kassahn KS, Gingras M-C, Muthuswamy LB, Johns AL, Miller DK, Wilson PJ, Patch A-M, Wu J, Chang DK, Cowley MJ, Gardiner BB (2012). Pancreatic cancer genomes reveal aberrations in axon guidance pathway genes. Nature.

[17] Tuveson DA, Shaw AT, Willis NA, Silver DP, Jackson EL, Chang S, Mercer KL, Grochow R, Hock H, Crowley D, Hingorani SR, Zaks T, King C (2004). Endogenous oncogenic K-rasG12D stimulates proliferation and widespread neoplastic and developmental defects. Cancer Cell.

[18] Stephen AG, Esposito D, Bagni RK, McCormick F (2014). Dragging ras back in the ring. Cancer Cell.

[19] Ebi H, Faber AC, Engelman JA, Yano S (2014). Not just gRASping at flaws: finding vulnerabilities to develop novel therapies for treating KRAS mutant cancers. Cancer Sci.

[20] Wolff NC, Pavía-Jiménez A, Tcheuyap VT, Alexander S, Vishwanath M, Christie A, Xie X-J, Williams NS, Kapur P, Posner B, McKay RM, Brugarolas J (2015). High-throughput simultaneous screen and counterscreen identifies homoharringtonine as synthetic lethal with von Hippel-Lindau loss in renal cell carcinoma. Oncotarget.

[21] Graab U, Hahn H, Fulda S (2015). Identification of a novel synthetic lethality of combined inhibition of hedgehog and PI3K signaling in rhabdomyosarcoma. Oncotarget.

[22] Samartzis EP, Gutsche K, Dedes KJ, Fink D, Stucki M, Imesch P (2014). Loss of ARID1A expression sensitizes cancer cells to PI3K- and AKT-inhibition. Oncotarget.

[23] Abbotts R, Jewell R, Nsengimana J, Maloney DJ, Simeonov A, Seedhouse C, Elliott F, Laye J, Walker C, Jadhav A, Grabowska A, Ball G, Patel PM (2014). Targeting human apurinic/apyrimidinic endonuclease 1 (APE1) in phosphatase and tensin homolog (PTEN) deficient melanoma cells for personalized therapy. Oncotarget.

[24] Hara T, Jones MF, Subramanian M, Li XL, Ou O, Zhu Y, Yang Y, Wakefield LM, Hussain SP, Gaedcke J, Ried T, Luo J, Caplen NJ (2014). Selective targeting of KRAS-mutant cells by miR-126 through repression of multiple genes essential for the survival of KRAS-mutant cells. Oncotarget.

[25] Wood M, Rawe M, Johansson G, Pang S, Soderquist RS, Patel A V, Nelson S, Seibel W, Ratner N, Sanchez Y (2011). Discovery of a small molecule targeting IRA2 deletion in budding yeast and neurofibromin loss in malignant peripheral nerve sheath tumor cells. Mol Cancer Ther.

[26] Parry D, Guzi T, Shanahan F, Davis N, Prabhavalkar D, Wiswell D, Seghezzi W, Paruch K, Dwyer MP, Doll R, Nomeir A, Windsor W, Fischmann T (2010). Dinaciclib (SCH 727965), a novel and potent cyclin-dependent kinase inhibitor. Mol Cancer Ther.

[27] Tong W-G, Chen R, Plunkett W, Siegel D, Sinha R, Harvey RD, Badros AZ, Popplewell L, Coutre S, Fox JA, Mahadocon K, Chen T, Kegley P (2010). Phase I and pharmacologic study of SNS-032, a potent and selective Cdk2, 7, and 9 inhibitor, in patients with advanced chronic lymphocytic leukemia and multiple myeloma. J Clin Oncol.

[28] Bausch D, Thomas S, Mino-Kenudson M, Fernández-del CC, Bauer TW, Williams M, Warshaw AL, Thayer SP, Kelly KA (2011). Plectin-1 as a novel biomarker for pancreatic cancer. Clin Cancer Res.

[29] Morton JP, Timpson P, Karim SA, Ridgway RA, Athineos D, Doyle B, Jamieson NB, Oien KA, Lowy AM, Brunton VG, Frame MC, Evans TRJ, Sansom OJ (2010). Mutant p53 drives metastasis and overcomes growth arrest/senescence in pancreatic cancer. Proc Natl Acad Sci U S A.

[30] Yachida S, White CM, Naito Y, Zhong Y, Brosnan JA, Macgregor-Das AM, Morgan RA, Saunders T, Laheru DA, Herman JM, Hruban RH, Klein AP, Jones S (2012). Clinical significance of the genetic landscape of pancreatic cancer and implications for identification of potential long-term survivors. Clin Cancer Res.

[31] Krasinskas AM, Chiosea SI, Pal T, Dacic S (2014). KRAS mutational analysis and immunohistochemical studies can help distinguish pancreatic metastases from primary lung adenocarcinomas. Mod Pathol.

[32] Ogura T, Yamao K, Hara K, Mizuno N, Hijioka S, Imaoka H, Sawaki A, Niwa Y, Tajika M, Kondo S, Tanaka T, Shimizu Y, Bhatia V (2013). Prognostic value of K-ras mutation status and subtypes in endoscopic ultrasound-guided fine-needle aspiration specimens from patients with unresectable pancreatic cancer. J Gastroenterol.

[33] Walters DK, Mercher T, Gu T-L, O'Hare T, Tyner JW, Loriaux M, Goss VL, Lee KA, Eide CA, Wong MJ, Stoffregen EP, McGreevey L, Nardone J (2006). Activating alleles of JAK3 in acute megakaryoblastic leukemia. Cancer Cell.

[34] Malinge S, Ragu C, Della-Valle V, Pisani D, Constantinescu SN, Perez C, Villeval J-L, Reinhardt D, Landman-Parker J, Michaux L, Dastugue N, Baruchel A, Vainchenker W (2008). Activating mutations in human acute megakaryoblastic leukemia. Blood.

[35] Ishimura N, Yamasawa K, Rumi MAK, Kadowaki Y, Ishihara S, Amano Y, Nio Y, Higami T, Kinoshita Y (2003). BRAF and K-ras gene mutations in human pancreatic cancers. Cancer Lett.

[36] Schultz NA, Roslind A, Christensen IJ, Horn T, Høgdall E, Pedersen LN, Kruhøffer M, Burcharth F, Wøjdemann M, Johansen JS (2012). Frequencies and prognostic role of KRAS and BRAF mutations in patients with localized pancreatic and ampullary adenocarcinomas. Pancreas.

[37] Calhoun ES, Jones JB, Ashfaq R, Adsay V, Baker SJ, Valentine V, Hempen PM, Hilgers W, Yeo CJ, Hruban RH, Kern SE (2003). BRAF and FBXW7 (CDC4, FBW7, AGO, SEL10) mutations in distinct subsets of pancreatic cancer: potential therapeutic targets. Am J Pathol.

[38] Daar AS, Fuggle S V, Fabre JW, Ting A, Morris PJ (1984). The detailed distribution of HLA-A, B, C antigens in normal human organs. Transplantation.

[39] Röther S, Strässer K (2007). The RNA polymerase II CTD kinase Ctk1 functions in translation elongation. Genes Dev.

[40] Wu Y, Chen C, Sun X, Shi X, Jin B, Ding K, Yeung SC, Pan J (2012). Cyclin-dependent kinase 7/9 inhibitor SNS-032 abrogates FIP1-like-1 platelet-derived growth factor receptor alpha and bcr-abl oncogene addiction in malignant hematologic cells. Clin Cancer Res.

[41] Hidalgo M, Bruckheimer E, Rajeshkumar N V, Garrido-Laguna I, De Oliveira E, Rubio-Viqueira B, Strawn S, Wick MJ, Martell J, Sidransky D (2011). A pilot clinical study of treatment guided by personalized tumorgrafts in patients with advanced cancer. Mol Cancer Ther.

[42] Thomas RM, Truty MJ, Kim M, Kang Y, Zhang R, Chatterjee D, Katz MH, Fleming JB (2015). The canary in the coal mine: the growth of patient-derived tumorgrafts in mice predicts clinical recurrence after surgical resection of pancreatic ductal adenocarcinoma. Ann Surg Oncol.

[43] Kleeff J, Reiser C, Hinz U, Bachmann J, Debus J, Jaeger D, Friess H, Büchler MW (2007). Surgery for recurrent pancreatic ductal adenocarcinoma. Ann Surg.

[44] Smeenk HG, Tran TCK, Erdmann J, van Eijck CHJ, Jeekel J (2005). Survival after surgical management of pancreatic adenocarcinoma: does curative and radical surgery truly exist?. Langenbecks Arch Surg.

[45] Gerlinger M, Rowan AJ, Horswell S, Larkin J, Endesfelder D, Gronroos E, Martinez P, Matthews N, Stewart A, Tarpey P, Varela I, Phillimore B, Begum S (2012). Intratumor heterogeneity and branched evolution revealed by multiregion sequencing. N Engl J Med.

[46] Bedard PL, Hansen AR, Ratain MJ, Siu LL (2013). Tumour heterogeneity in the clinic. Nature.

[47] Nakamura T, Kuwai T, Kitadai Y, Sasaki T, Fan D, Coombes KR, Kim S-J, Fidler IJ (2007). Zonal heterogeneity for gene expression in human pancreatic carcinoma. Cancer Res.

[48] Chang KJ, Nguyen P, Erickson RA, Durbin TE, Katz KD (1997). The clinical utility of endoscopic ultrasound–guided fine-needle aspiration in the diagnosisand staging of pancreatic carcinoma. Gastrointest Endosc.

[49] Voss M (2000). Value of endoscopic ultrasound guided fine needle aspiration biopsy in the diagnosis of solid pancreatic masses. Gut.

[50] Harewood GC, Wiersema MJ (2002). Endosonography-guided fine needle aspiration biopsy in the evaluation of pancreatic masses. Am J Gastroenterol.

[51] Tignanelli CJ, Herrera Loeza SG, Yeh JJ (2014). KRAS and PIK3CA mutation frequencies in patient-derived xenograft models of pancreatic and colorectal cancer are reflective of patient tumors and stable across passages. Am Surg.

[52] Monsma DJ, Cherba DM, Richardson PJ, Vance S, Rangarajan S, Dylewski D, Eugster E, Scott SB, Beuschel NL, Davidson PJ, Axtell R, Mitchell D, Lester EP (2014). Using a rhabdomyosarcoma patient-derived xenograft to examine precision medicine approaches and model acquired resistance. Pediatr Blood Cancer.

[53] Malaney P, Nicosia S V, Davé V (2014). One mouse, one patient paradigm: New avatars of personalized cancer therapy. Cancer Lett.

[54] Schindelin J, Arganda-Carreras I, Frise E, Kaynig V, Longair M, Pietzsch T, Preibisch S, Rueden C, Saalfeld S, Schmid B, Tinevez J-Y, White DJ, Hartenstein V (2012). Fiji: an open-source platform for biological-image analysis. Nat Methods.

[55] Burgess A, Vigneron S, Brioudes E, Labbé J-C, Lorca T, Castro A (2010). Loss of human Greatwall results in G2 arrest and multiple mitotic defects due to deregulation of the cyclin B-Cdc2/PP2A balance. Proc Natl Acad Sci U S A.

[56] Potapova TA, Sivakumar S, Flynn JN, Li R, Gorbsky GJ (2011). Mitotic progression becomes irreversible in prometaphase and collapses when Wee1 and Cdc25 are inhibited. Mol Biol Cell.

[57] Greene C, Allaway R, Kacsoh B (2015). Data and analytical code for in vitro and PDX analysis.

[58] Oliner K, Juan T, Suggs S, Wolf M, Sarosi I, Freeman DJ, Gyuris T, Baron W, Bakker A, Parker A, Patterson SD (2010). A comparability study of 5 commercial KRAS tests. Diagn Pathol.

[59] R Core Team (2012). R: A language and environment for statistical computing.

[60] Wickham H (2009). ggplot2: elegant graphics for data analysis.

