# Supplementary material for: Genomic characterization of patient-derived xenograft models established from fine needle aspirate biopsies of a primary pancreatic ductal adenocarcinoma and from patient-matched metastatic sites
Source: Oncotarget. 2016 Feb 25;7(13):17087–102. doi: 10.18632/oncotarget.7718 (PMC4941373; doi:10.18632/oncotarget.7718)
Supplement: Supplementary file 1 [file oncotarget-07-17087-s001.pdf]

SUPPLEMENT: CLINICAL COURSE OF PATIENT DESIGNATED 008

Patient 008 is a middle-aged individual who presented to our institution with jaundice, weight loss and diarrhea. A 3-cm mass in the head of the pancreas was noted on CT imaging without radiographic evidence for metastatic disease. The patient underwent EUS/FNA, and a diagnosis of PDAC was established. The 5th pass of FNA specimen acquisition was obtained for PDX engraftment. Two months post-FNA, the patient started systemic chemotherapy with gemcitabine, docetaxel and capecitabine per outside cancer center consultation. This was ultimately complicated by gastrointestinal bleeding and a bowel obstruction. Five months post-FNA, this patient presented to our institution with a symptomatic bowel obstruction and surgical oncology was consulted and surgery was recommended. At the time of exploration, several small, < 5 mm liver nodules and multiple similar sized peritoneal nodules were identified. Intraoperative biopsy confirmed metastatic disease consistent with the patient's pancreatic primary adenocarcinoma. PDX models were established from two specimens, one from a peritoneal metastasis (M1), and one from a liver metastasis (M2).

SUPPLEMENTARY FIGURES

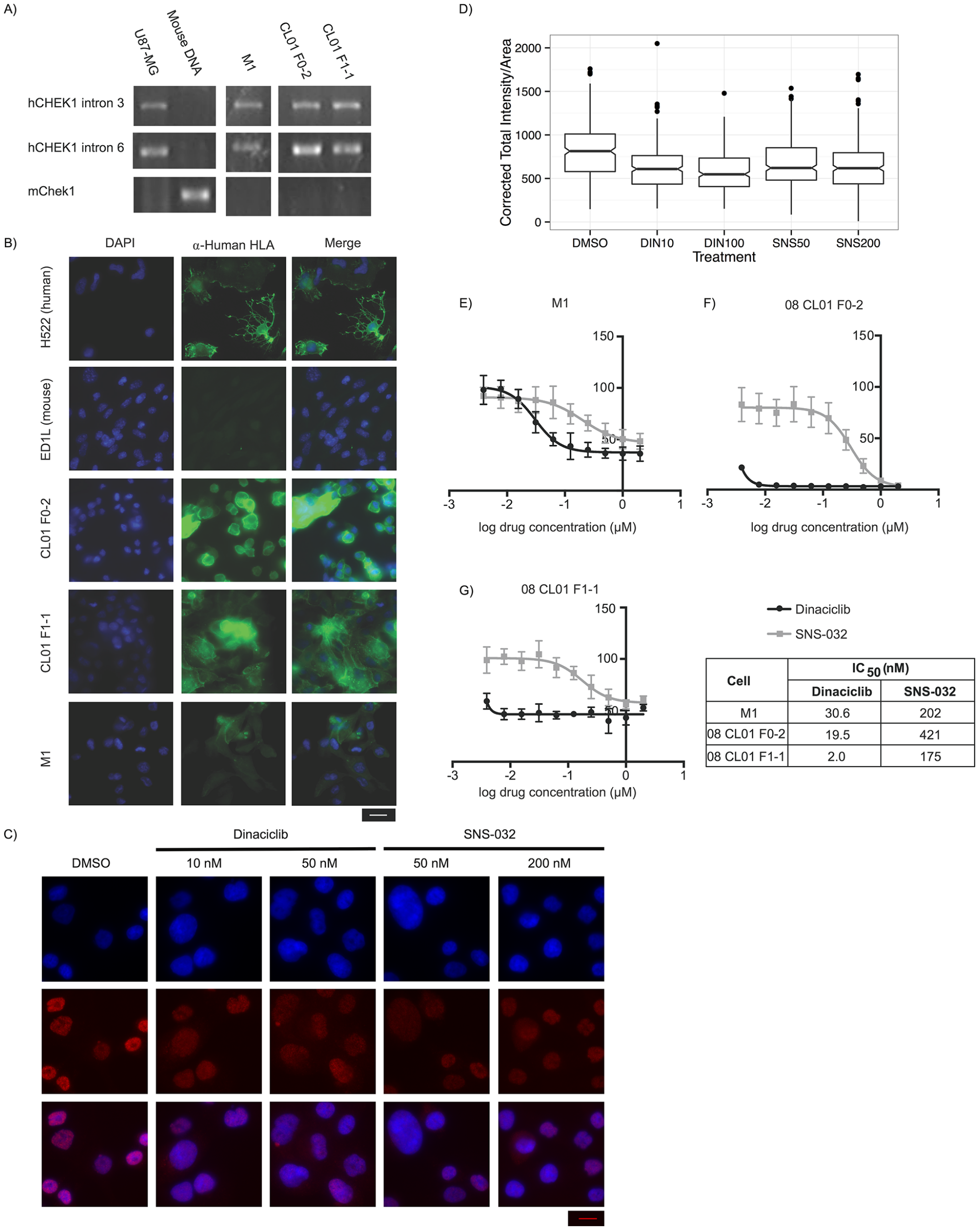

Cell	IC 50 (nM)	
	Dinaciclib	SNS-032
M1	30.6	202
08 CL01 F0-2	19.5	421
08 CL01 F1-1	2.0	175

Supplementary Figure S1: Explant cells from PDX model are sensitive to CDK9 inhibitors. A. Human and mouse specific *CHEK1* primers PCR were used to identify mouse and human content in PDX explant cells (same controls as Figure 4A). PDX explant cells from a peritoneal metastasis (M1) and subsequent passages of that PDX model (08 CL01 F0–2 and 08 CL01 F1–1) had human *CHEK1* DNA but did not have mouse *Chek1* DNA. **B.** α-Human HLA A, B and C protein in PDX explant cells was evaluated by immunofluorescence as a measure of human content. All explant cells evaluated were found to be α-Human HLA A, B and/or C positive (same controls as Figure 4B). **C-D.** PDX explant cells from the peritoneal metastasis (M1) treated for 24 h with the CDK9 inhibitors dinaciclib (10 nM and 200 nM) and SNS-032 (50 nM and 200 nM) have reduced Ser2/5 phosphorylation of the CTD of the large subunit of RNAPII as measured by total fluorescent intensity per unit area of cells treated with dinaciclib and SNS-032 and stained for p-Ser2/5 RNAPII CTD. **E-H.** Dinaciclib and SNS-032 inhibit the growth of explant cells during a 3-day treatment. Plotted points represent the average fluorescence measurement from 4 wells; error bars are standard error of the mean.

A)

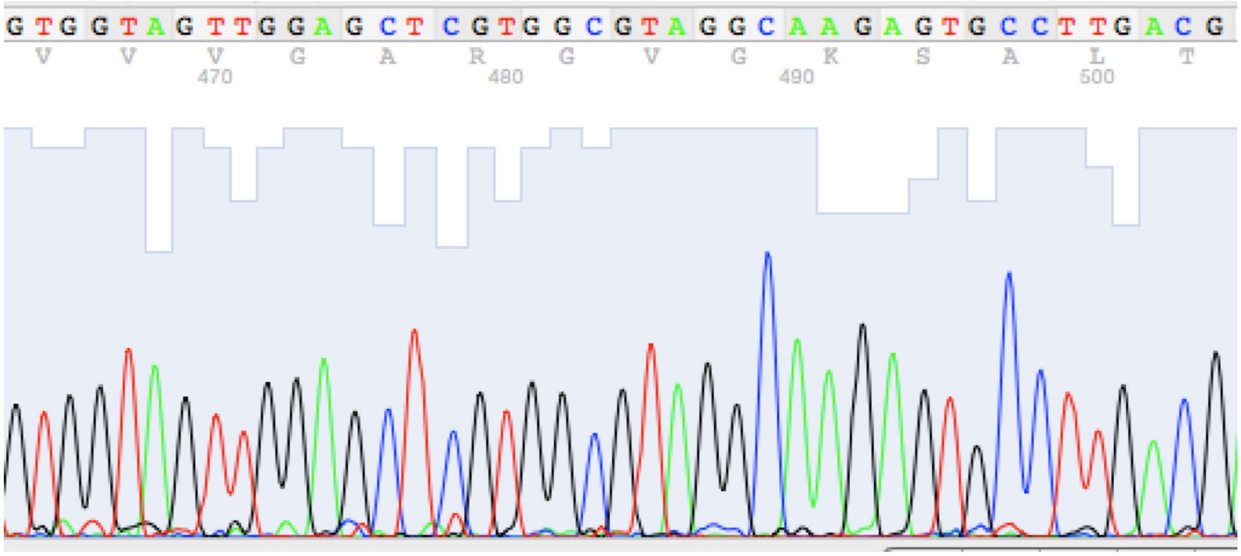

B)

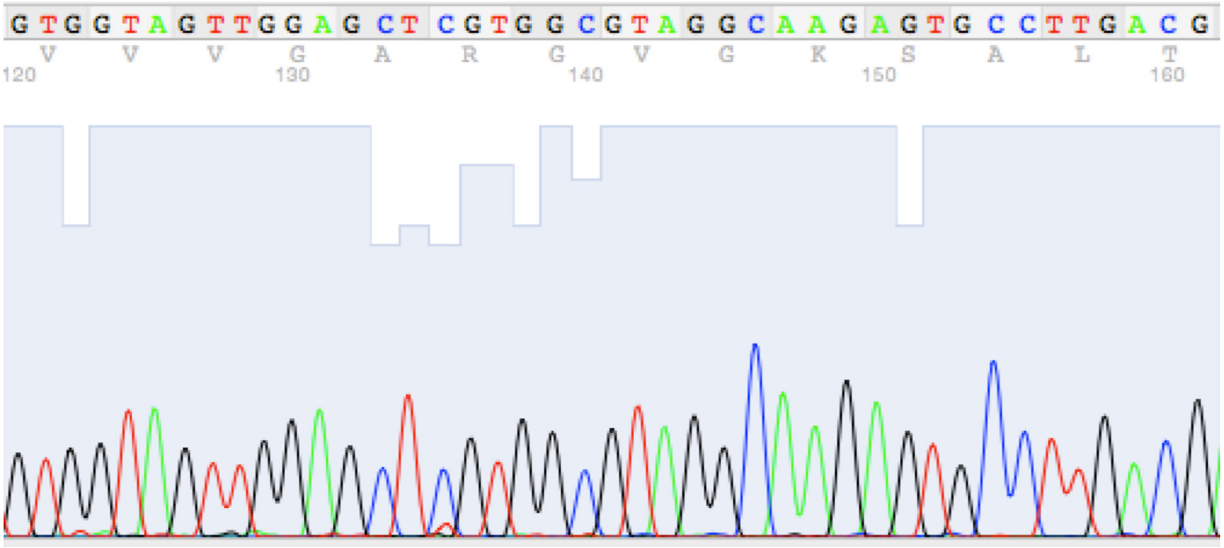

Supplementary Figure S2: Explant cells derived from M1 and M2 tumors have a *KRAS* G12R mutation present. Sanger sequencing was performed to confirm that explant cells from M1 **A.** and M2 **B.** PDX tumors maintained the *KRAS* G12R mutation.
